# Lung Function Reserve and Physical Function in Healthy Older Adults: Findings from BLSA

**DOI:** 10.1093/geroni/igab046.3504

**Published:** 2021-12-17

**Authors:** Joey Saavedra, Ajoy Karikkineth, Luigi Ferrucci, Eleanor Simonsick

**Affiliations:** 1 Iowa State University, Ames, Iowa, United States; 2 National Institute on Aging, Baltimore, Maryland, United States; 3 National Instute on Aging/NIH, Baltimore, Maryland, United States

## Abstract

Forced Expiratory Volume in 1-second (FEV1) that falls below the lower limit of normal (LLN) is a well-established correlate of functional limitation and disability. However, less is known about the functional implications of gradations of lung function above the LLN. We examined the cross-sectional association between gradations of healthy lung function and usual gait speed, reported walking ability, and fast 400m walk performance in 750 persons (50.7% men) aged 55-95 free from respiratory disease and mobility limitations, participating in the Baltimore Longitudinal Study of Aging (BLSA). The 2012 Global Lung Initiative (GLI) reference equations were used to calculate FEV1 Z-scores, with healthy lung function categorized as follows: -1.6 < Z ≤ -1.0 (pre-clinical), -1.0 < Z ≤ -0.3 (low normal), -0.3 < Z ≤ 0.3 (normal), 0.3 < Z ≤ 1.0 (high-normal), and Z > 1.0 (high). Associations between gradations of healthy lung function and physical function were evaluated using multivariate linear regression, adjusting for age, sex, height, weight, and waist circumference. Compared to the ‘pre-clinical’ category, the difference in 400m walk time was 0.71 (p>.05), -6.60 (p>.05), -12.21 (p<0.05), and -15.52 (p<0.01) seconds for the ‘low normal’, ‘normal’, ‘high-normal’, and ‘high’ categories, respectively. No associations between gradations of healthy lung function and normal gait speed or walking ability were found (p>0.05). Higher levels of lung function reserve are associated with better 400m walking performance, thus efforts to promote and/or reduce loss of lung function reserve may help individuals maintain high functional capacity in later life.

